# An Unusual Gross Appearance of Vulval Tuberculosis Masquerading as Tumor

**DOI:** 10.1155/2014/815401

**Published:** 2014-09-14

**Authors:** Surekha U. Arakeri, Prachi Sinkar

**Affiliations:** Shri B.M. Patil Medical College Hospital & Research Centre, BLDE University, Bijapur 586103, India

## Abstract

Tuberculosis of the vulva is very rare. It is found in about 0.2% of the cases of genital tract tuberculosis. It usually presents as small shallow ulcers and multiple sinus tracts or rarely as elephantiasis of vulva. Except for very rare cases of primary tuberculosis in the vulva, it is usually associated with tuberculosis elsewhere in the body leading to secondary tuberculosis. Here, we report a case of secondary vulval tuberculosis which presented as a vulval mass in a 40-year-old female patient. The rarity of this presentation in the female genital tract is emphasized.

## 1. Introduction

Tuberculosis is a major health problem in India. Tuberculosis of genital tract constitutes about 0.2% to 2% of all gynecological cases. It is usually asymptomatic and mainly affects young women in the reproductive age group. Many authors opined that there may be hormone dependence of infection, as 80% of cases of tuberculosis of genital tract occur in the reproductive age group [1–3]. It frequently affects the upper genital tract generally affecting fallopian tubes and endometrium and is presented clinically with varying symptoms and signs spanning from fertility problems to pregnancy complications including pregnancy losses, abnormal vaginal bleeding, vaginal discharge, menstrual irregularities, abdominal pain, or constitutional symptoms [4]. Vulval tuberculosis is rare and is diagnosed in about 0.2% of the cases of genital tract tuberculosis [2]. Tuberculosis of vulva can occur at any age. It has been reported in patients varying between the age group of 7 months to 85 years. It usually presents as ulcer and multiple sinuses with discharge, or rarely as elephantiasis of vulva. Due to bizarre clinical presentation, clinical diagnosis of vulval tuberculosis is a challenging task to the clinicians [4].

## 2. Case Report

A female patient aged 40 years old presented with complaints of vulval growth, multiple nodules with opening of sinuses, and yellowish white discharge since two years. Culture of discharge material from sinus was positive for staphylococcus bacilli. Based on these complaints and culture findings, patient was administered several courses of antibiotics. However, patient did not respond to the antibiotic treatment. Her past clinical history revealed that she had pulmonary tuberculosis since 15 years, for which she had taken an incomplete course of antitubercular treatment. She denied any premarital or extramarital sexual relationship. Her menstrual cycles were regular. Her obstetric history was normal with two living children. On examination, hypertrophied cauliflower like mass covering the entire labia majora was noted. Both sided inguinal lymph nodes were discrete and nontender. Ultrasonography of lower abdomen revealed no abnormal findings in bilateral fallopian tubes, ovaries, or uterus. Routine blood and biochemical investigations of patient were within normal limits. Partial vulvectomy procedure was done and the specimen was sent for histopathology study. 

**Figure 1:**
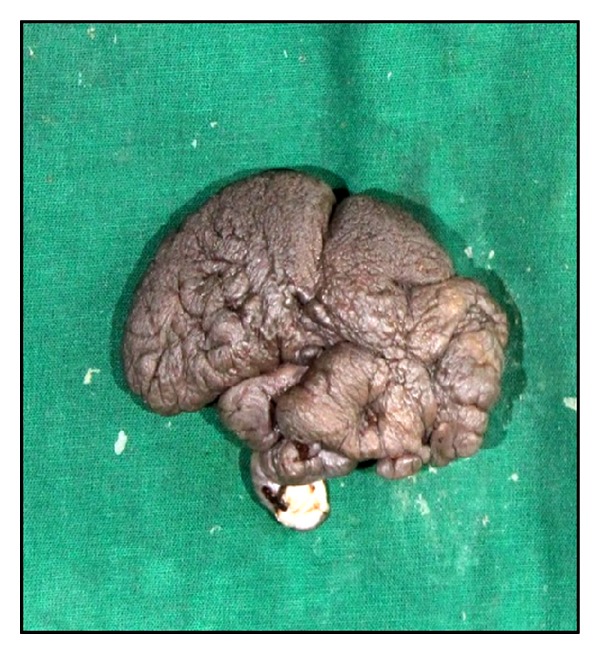
Gross photograph of partial vulvectomy specimen showing nodular skin surface.

**Figure 2:**
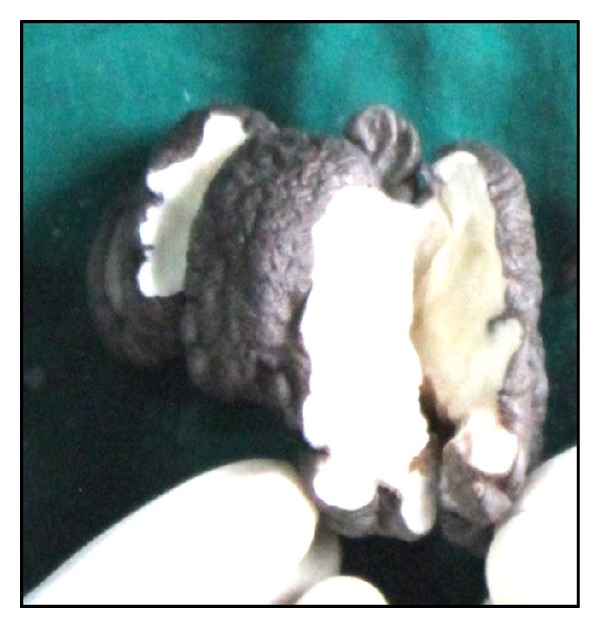
Cut surface showing irregular, pale-white solid appearance with foci of capillary sized blood vessels.

*Macroscopy.* A skin covered tissue mass measuring 9 × 4 × 2.5 cm was received. Skin surface was nodular with openings of sinuses over some nodules. Cut surface was pale-white glistening and predominantly solid in appearance with tiny foci of capillary sized blood vessels filled with blood (Figures 1 and 2). 

**Figure 3:**
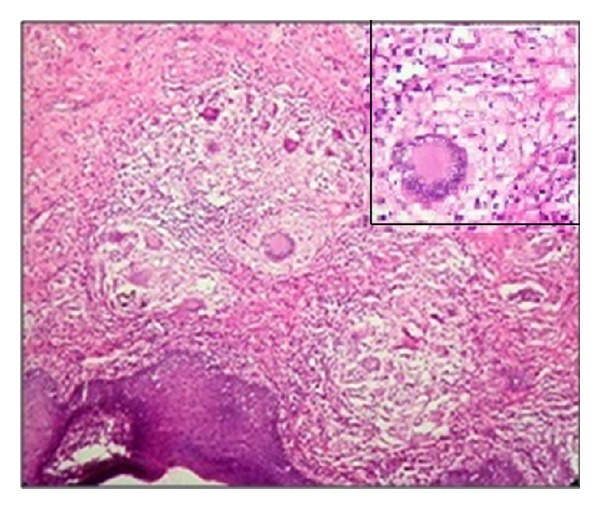
Microphotograph showing granulomas and lymphocytic infiltration (H&E 10x). Inset granuloma (H&E 40x).

**Figure 4:**
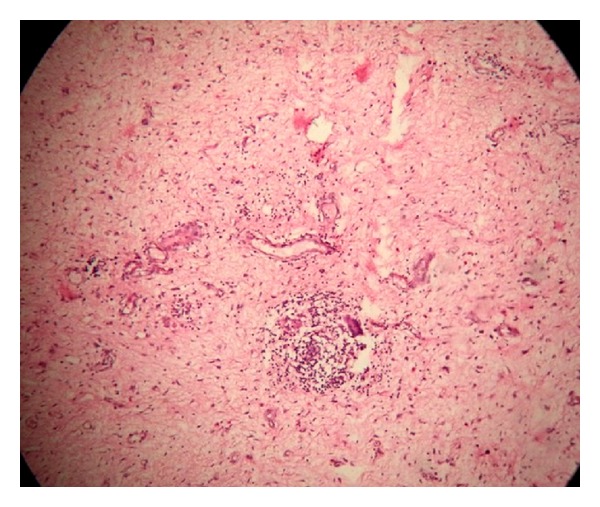
Microphotograph showing myxoid area, inflammatory change, and granuloma (H&E 10x).

*Microscopy.* Pseudoepitheliomatous hyperplasia of epidermis was noted in the microscopy. Dermis was loose edematous and showed fibrocollagenous and fibrovascular tissue with focal and diffuse mononuclear cell infiltration. Focal myxoid areas and few granulomas formed of epithelioid cells and Langhans giant cells surrounded by lymphocytes, plasma cells, and histiocytes were also noted (Figures 3 and 4). ZN stain for AFB was negative. Based on these findings, an impression of tuberculosis of vulva was given.

## 3. Discussion

Tubercular lesion of vulva usually presents as small shallow ulcers and multiple sinus tracts. Also it rarely presents as elephantiasis of vulva or hypertrophic vulval tuberculosis [4]. In the present case, vulval tuberculosis is presented as vulval mass for which partial vulvectomy was done. Gross examination of vulvectomy specimen showed pale white solid glistening appearance with tiny cystic blood filled spaces on cut surface masquerading as angiomyxoma. Circumscribed mass was not seen in this case, which is one of the characteristic gross appearances of angiomyxoma. Microscopy in this case revealed edema and inflammatory changes, which mostly represents inflammatory change resulting from lymphatic obstruction as stated by Tiwari et al. [4] in their case report of hypertrophic tuberculosis of vulva. The diagnosis of vulvovaginal tuberculosis is usually made by histological examination of vulvovaginal biopsy specimen. Isolation of mycobacterium bacilli is the gold standard for diagnosis of tuberculosis. However, bacilli are very rarely found in female genital tract tuberculosis even with fluorescent techniques. Most authors agree that histological examination is one of the most useful means of establishing diagnosis of genital tract tuberculosis and presence of typical granulomas is sufficient for confirming the diagnosis. The differential diagnosis for granulomatous disease includes amoebiasis, schistosomiasis, brucellosis, tularemia, sarcoidosis, and foreign body reaction. Vulval tuberculosis is routinely treated by clinicians as a sexually transmitted disease, because of the rarity of tuberculosis involving the vulva. Only a high index of suspicion leads to diagnosis of tuberculosis [5–7].

Hematogenous or lymphatic dissemination from an active site of infection is the most common mode of spread of tubercular infection in female genital tract. Infection may also spread from contiguous intra-abdominal sites through fallopian tubes to female genital tract, or it can also be primary tuberculosis of vulva by direct inoculation of bacilli by a male partner suffering from tubercular lesion in epididymis and seminal vesicles. Tubercular vulvitis is usually associated with tuberculosis of upper genital tract. Except for very rare cases of primary inoculation in the vulva, vulval tuberculosis is usually associated with tuberculosis elsewhere in the body [5, 6]. In the present case there was no evidence of tuberculosis in the upper genital tract. The present case is considered as secondary tuberculosis of vulva as patient had history of pulmonary tuberculosis 15 years ago with partial treatment. The patient was advised antitubercular treatment again, and after about two-month follow-up it was observed that there were no sinuses or discharge.

## 4. Conclusion

Detailed and thorough medical history recording, proper clinical examination, and presence of granulomatous reaction in microscopic examination can act as reliable diagnostic tools in detecting and managing vulval tuberculosis in patients presenting with unusual findings and help in prevention of patient undergoing unnecessary surgical interventions.
